# Catalysis of transthiolacylation in the active centers of dihydrolipoamide acyltransacetylase components of 2‐oxo acid dehydrogenase complexes

**DOI:** 10.1002/2211-5463.12431

**Published:** 2018-06-04

**Authors:** Joydeep Chakraborty, Natalia S. Nemeria, Edgardo Farinas, Frank Jordan

**Affiliations:** ^1^ Department of Chemistry and Environmental Science New Jersey Institute of Technology Newark NJ USA; ^2^ Department of Chemistry Rutgers University Newark NJ USA

**Keywords:** 2‐oxo acid dehydrogenase complexes, dihydrolipoamide acyltransferase E2 component, oxyanionic intermediate stabilization, saturation mutagenesis, transthioesterification

## Abstract

The *Escherichia coli* 2‐oxoglutarate dehydrogenase complex (OGDHc) comprises multiple copies of three enzymes—E1o, E2o, and E3—and transthioesterification takes place within the catalytic domain of E2o. The succinyl group from the thiol ester of S8‐succinyldihydrolipoyl‐E2o is transferred to the thiol group of coenzyme A (CoA), forming the all‐important succinyl‐CoA. Here, we report mechanistic studies of enzymatic transthioesterification on OGDHc. Evidence is provided for the importance of His375 and Asp374 in E2o for the succinyl transfer reaction. The magnitude of the rate acceleration provided by these residues (54‐fold from each with alanine substitution) suggests a role in stabilization of the symmetrical tetrahedral oxyanionic intermediate by formation of two hydrogen bonds, rather than in acid–base catalysis. Further evidence ruling out a role in acid–base catalysis is provided by site‐saturation mutagenesis studies at His375 (His375Trp substitution with little penalty) and substitutions to other potential hydrogen bond participants at Asp374. Taking into account that the rate constant for reductive succinylation of the E2o lipoyl domain (LDo) by E1o and 2‐oxoglutarate (99 s^−1^) was approximately twofold larger than the rate constant for *k*_cat_ of 48 s^−1^ for the overall reaction (NADH production), it could be concluded that succinyl transfer to CoA and release of succinyl‐CoA, rather than reductive succinylation, is the rate‐limiting step. The results suggest a revised mechanism of catalysis for acyl transfer in the superfamily of 2‐oxo acid dehydrogenase complexes, thus provide fundamental information regarding acyl‐CoA formation, so important for several biological processes including post‐translational succinylation of protein lysines.

**Enzymes:**

2‐oxoglutarate dehydrogenase (http://www.chem.qmul.ac.uk/iubmb/enzyme/EC1/2/4/2.html); dihydrolipoamide succinyltransferase (http://www.chem.qmul.ac.uk/iubmb/enzyme/EC2/3/1/61.html); dihydrolipoamide dehydrogenase (http://www.chem.qmul.ac.uk/iubmb/enzyme/EC1/8/1/4.html); pyruvate dehydrogenase (http://www.chem.qmul.ac.uk/iubmb/enzyme/EC1/2/4/1.html); dihydrolipoamide acetyltransferase (http://www.chem.qmul.ac.uk/iubmb/enzyme/EC2/3/1/12.html).

**Abbreviations**
acetyl‐CoA: acetyl‐coenzyme A
CDo: E2o catalytic domain
E1o: 2‐oxoglutarate dehydrogenase, the first component of the OGDHc
E1p: pyruvate dehydrogenase
E2o^1–176^: a didomain, comprising the LDo and linker including the PSBDo
E2o: dihydrolipoamide succinyltransferase, the second component of the OGDHc
E2p: dihydrolipoamide acetyltransferase
E3: dihydrolipoamide dehydrogenase, the third component of the OGDHc
FT‐MS: fourier transform mass spectrometry with electrospray ionization sampling method
LDo: E2o lipoyl domain
LDp: E2p hybrid lipoyl domain, where residues 1–33 are from the N‐terminal end of the first lipoyl domain and residues 34–85 are from the C‐terminal end of the third lipoyl domain of the wild‐type three lipoyl domain E2p
*o*: 2‐oxoglutarate dehydrogenase complex origin
OA: 2‐oxoadipate succinyl‐CoAsuccinyl‐coenzyme A
OG: 2‐oxoglutarate
OGDHc: *Escherichia coli* 2‐oxoglutarate dehydrogenase multienzyme complex
OV: 2‐oxovalerate
PDHc: *Escherichia coli* pyruvate dehydrogenase multienzyme complex
*P*: pyruvate dehydrogenase complex origin
PSBDo: the peripheral subunit binding domain of E2o
TCEP: tris(2‐carboxyethyl)phosphine
ThDP: thiamin diphosphate

The 2‐oxoglutarate dehydrogenase complex (OGDHc, also known as α‐ketoglutarate dehydrogenase complex) catalyzes one of the rate‐limiting steps in the tricarboxylic acid cycle (TCA), which is a common pathway for oxidation of fuel molecules, including carbohydrates, fatty acids, and amino acids. The OGDHc is a multienzyme complex composed of multiple copies of three component enzymes: a thiamin diphosphate (ThDP)‐dependent 2‐oxoglutarate dehydrogenase (E1o; http://www.chem.qmul.ac.uk/iubmb/enzyme/EC1/2/4/2.html), dihydrolipoyl succinyltransferase (E2o; http://www.chem.qmul.ac.uk/iubmb/enzyme/EC2/3/1/61.html), and dihydrolipoyl dehydrogenase (E3; http://www.chem.qmul.ac.uk/iubmb/enzyme/EC1/8/1/4.html) (Fig. 1) 1, 2, 3. The E1o and E2o carry out the principal reactions for succinyl‐CoA formation, whereas E3 re‐oxidizes dihydrolipoamide‐E2o to lipoamide‐E2o, the redox cofactor covalently amidated onto a lysine residue on the E2o component.

**Figure 1:**
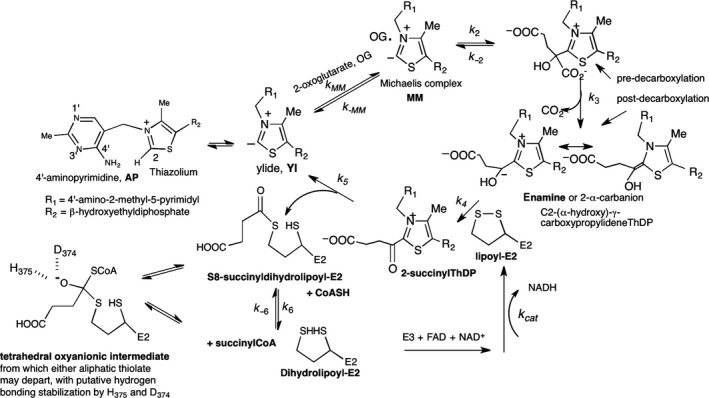
Mechanism of 2‐oxoglutarate dehydrogenase complex and the putative oxyanionic tetrahedral intermediate suggesting the role of His375 and Asp 374 in E2o active center.

Recent studies revealed multiple roles for the mitochondrial OGDHc, and their better understanding could be important for bringing new insight into human diseases. The unique property of the mitochondrial OGDHc to produce the reactive oxygen species superoxide and hydrogen peroxide (H_2_O_2_) from its substrate 2‐oxoglutarate (OG) had been attributed earlier to the flavin cofactor tightly bound to the E3 component 4, 5. Our studies revealed that human E1o (E1o‐h) can produce the ThDP‐enamine radical and superoxide anion from OG and from the next higher homologue 2‐oxoadipate (OA) by one‐electron oxidation of the ThDP‐enamine intermediate with dioxygen 6, 7. The efficiency of superoxide/H_2_O_2_ production by OGDHc was 7 times larger from OA than from OG making the OGDHc one of the important reactive oxygen species producers among 2‐oxo acid dehydrogenase complexes in mitochondria 5, 7.

In a role different from its role in the TCA cycle, the OGDHc could also serve as a producer of succinyl groups in neurons and neuronal cell lines for reversible post‐translational modification of the cytosolic and mitochondrial enzymes, including those from the TCA, by succinylation, hence playing a role in neurodegenerative diseases 8. The post‐translational modification of histone proteins by succinylation is regarded as very important because it can directly regulate gene expression 9, 10. Recently, evidence of the interaction between the nuclear OGDHc and lysine acetyltransferase 2A displayed a role of lysine acetyltransferase as a carrier of succinyl groups produced by nuclear OGDHc for direct histone H3 succinylation 9.

Our own recent studies suggested that human 2‐oxoadipate dehydrogenase (hE1a, also known as *DHTKD1*‐encoded protein), which is involved in the oxidative decarboxylation of 2‐oxoadipate to glutaryl‐CoA on the final degradative pathway of l‐lysine, l‐hydroxylysine, and l‐tryptophan, has recruited the hE2o and hE3 components of the OGDHc for its function 11. In other words, the hE2o could serve as a source of both succinyl‐CoA and glutaryl‐CoA in mitochondria and could be linked to the lysine post‐translational modification by glutarylation earlier reported 12.

The enzymatic mechanism being studied in this research is responsible for synthesis of important acyl‐CoA metabolites involved in post‐translational modification of proteins. The results provide important baseline residue‐specific contribution to catalysis of succinyl‐CoA formation in the active center of the *Escherichia coli* E2o that would be applicable to all E2o components due to high sequence identities reported for all E2o core/catalytic domains 13, 14, 15, 16, 17, 18, 19. The major goal of this study was to address the role of the highly conserved His375 in the active center of the *E. coli* E2o. The His375Ala substitution was found to decrease the catalytic efficiency (*k*_cat_ */K*_m,OG_) by 54‐fold compared to unsubstituted E2o. This value was approximately 10 000 times smaller than that determined on Ala substitution of the corresponding His195 in chloramphenicol acetyltransferase (CAT*, k*_cat_ */K*_m_ **= 53 × 10^−4^‐fold smaller for the His195Ala variant than wild‐type) 20, placing in doubt the earlier deduction of an acid–base catalyst role of His375, assigned by comparison with CAT 21, 22. To gain more insight regarding the function of His375, site‐saturation mutagenesis was carried out at this residue, whose results suggested that the His375Trp and His375Gly substitutions at E2o were acceptable, reducing the catalytic efficiency only by two‐ and fivefold, respectively. Similarly, the Asp374Asn substitution in the E2o active center (10 times less efficient variant) rescued some of the activity displayed by the Asp374Ala substitution (54‐times less efficient variant), consistent with participation of Asp374 in hydrogen bond formation. The totality of our studies suggested that there is no proton transfer in the rate‐limiting step in which both His375 and Asp374 participate; rather, they may stabilize by hydrogen bonds a transition state, probably resembling a tetrahedral oxyanionic intermediate.

## Materials and methods

### Reagents

Dithiothreitol (DTT), 2‐oxoglutarate, 2‐oxovalerate, 2‐oxoadipate, and pyruvate were from Sigma‐Aldrich (St. Louis, MO, USA). NADH, CoA, isopropyl‐β‐D‐thiogalactopyranoside (IPTG), DNase I, micrococcal nuclease, DL‐α‐lipoic acid, and ThDP were from Affymetrix (Cleveland, OH, USA). Protease inhibitor cocktail tablets were from Roche Diagnostics GmbH (Mannheim, Germany). Ni‐Sepharose 6 Fast Flow and HiPrep™ 26/60 Sephacryl™ S‐300 HR column were from GE Healthcare (Pittsburgh, PA, USA). QuikChange Site‐Directed Mutagenesis Kit was from Agilent Technologies (Santa Clara, CA, USA). Primers for site‐directed mutagenesis were from Fisher Scientific (Pittsburgh, PA, USA). *Escherichia coli* strain JW0715 containing the pCA24N plasmid encoding the E1o component and JW0716 containing pCA24N plasmid encoding the E2o component were obtained from National Bio Resource Project (NIG, Japan). AG1 cells (Agilent Technologies) were used as host cells.

### Protein expression and purification

#### Expression and purification of E1o

Expression and purification of E1o was as reported earlier with some modifications 23. The E1o component was purified using a Ni‐Sepharose 6 Fast Flow column equilibrated with 20 mm KH_2_PO_4_ (pH 7.5) containing 0.20 m KCl, 0.20 mm ThDP, 1.0 mm MgCl_2_, 1.0 mm benzamidine·HCl (buffer A), and 30 mm imidazole. After the protein was applied to the column, the column was washed with 500 mL of 30 mm imidazole and then with 500 mL of 50 mm imidazole, both in buffer A. The E1o was eluted with 150 mm imidazole in buffer A and was dialyzed against 2000 mL of 20 mm KH_2_PO_4_ (pH 7.5) containing 0.35 mm KCl, 0.20 mm ThDP, 1.0 mm MgCl_2_, and 1.0 mm benzamidine·HCl for 15 h at 4 °C. For E1o storage, buffer was exchanged to 20 mm KH_2_PO_4_ (pH 7.5) containing 0.20 m KCL, 0.20 mm ThDP, and 1.0 mm MgCl_2_ by ultrafiltration using a concentrating unit with a 30‐kDa cutoff. The E1o was stored at −80 °C.

#### Expression and purification of the E2o and its variants

Expression and purification of E2o was as reported earlier for human E2p 24 with some modifications. AG1 cells were grown in LB medium supplemented with 35 μg·mL^−1^ of chloramphenicol, and protein expression was induced by 0.50 mm IPTG for 5 h at 37 °C. Harvested cells were resuspended in 20 mm KH_2_PO_4_ (pH 7.5) containing 0.30 m NaCl, 1 mm benzamidine·HCl, 1 mm DTT, and two protease inhibitor cocktail tablets. The resulting cell suspension was incubated with lysozyme (0.60 mg·mL^−1^) for 20 min on ice. Next, MgCl_2_ (5 mm) and 1000 U each of DNase I and micrococcal nuclease were added and the cells were incubated for an additional 20 min on ice. Cells were then disrupted by ultrasonication at a setting of 6 using 10‐s pulse ‘on’ and 30‐s pulse ‘off ‘for total time of 5 min. PEG‐8000 (50% w/v) was added dropwise to the clarified lysate to 6% (v/v), and the precipitated pellet was dissolved in 20 mm KH_2_PO_4_ (pH 7.5) containing 0.30 m NaCl, 1 mm DTT, 1.0 mm benzamidine·HCl, and 1 mm EDTA. The E2o was purified using a Sephacryl S‐300 high‐resolution size‐exclusion column equilibrated with 20 mm KH_2_PO_4_ (pH 7.5) containing 0.30 m NaCl, 1 mm DTT, 1.0 mm benzamidine·HCl, and 1 mm EDTA. Fractions containing E2o according to PAGE were collected, and E2o was precipitated by ultracentrifugation for 4.0 h at 140 000 *g*. The pellet was dissolved in 20 mm KH_2_PO_4_ (pH 7.5) containing 0.40 m NaCl, 0.50 mm EDTA, 1.0 mm DTT, and 1.0 mm benzamidine·HCl for 15 h at 4 °C. The clarified E2o was stored at −80 °C.

The E2o variants with Thr323Ala, Thr323Ser, Asp374Ala, Asp374Asn, His375Ala, His375Cys, His375Asn, Arg376Ala, and Asp379Ala substitutions were created using the pCA24N‐E2o plasmid as a template and two synthetic oligonucleotide primers complementary to the opposite strands of the DNA with the QuikChange Site‐Directed Mutagenesis Kit and protocol supplied by the manufacturer (Stratagene, La Jolla, CA, USA). The following oligonucleotide primers and their complements were used (mismatched bases are underlined, and mutated codons are shown in boldface type) as listed in Table 1.

**Table 1:** Primer table

Substitution	Primer sequence
Thr323Ala	5′‐AACTTCACCATC**GCG**AACGGTGGTGTGTTCGGTTCC**‐**3**′**
Thr323Ser	5′‐AACTTCACCATCA**G**CAACGGTGGTGTGTTCGGTTCC‐3′
Asp374Ala	5′‐CTGGCGCTGTCCTAC**GCG**CACCGTCTGATC‐3′
Asp374Asn	5′‐GGCGCTGTCCTAC**A**A**C**CACCGTCTG‐3′
His375Ala	5′‐GCGCTGTCCTACGAT**GCG**CGTCTGATCGAT‐3′
His375Cys	5′‐GCGCTGTCCTACGAT**TG**CCGTCTGATCGAT‐3′
His375Asn	5′‐GCTGTCCTACGAT**A**ACCGTCTGATCG‐3′
Arg376Ala	5′‐TCCTACGATCAC**GCG**CTGATCGATGGTCGC‐3′
Asp379Ala	5′‐CACCGTCTGATC**GCG**GGTCGCGAATCCGTG‐3′
His375X	5′‐GGCGCTGTCCTACGAT**NNS**CGTCTGATC**G‐**3′

N = A/T/G/C and S = G/C.

The presence of substitutions was verified by DNA sequencing with specific primers at the Molecular Resource Facility of Rutgers New Jersey Medical School.

#### Expression and purification of LDo and E2o^1–176^ didomain

Expression and purification of the E2o lipoyl domain (LDo, residues 1–105 in E2o) and E2o^1–176^ didomain was as reported earlier 15, 25 for *E. coli* LDp and E2p^1–190^. To ensure full lipoylation of the LDo and the E2o^1–176^ didomain, these E2o‐derived domains were lipoylated *in vitro* by an *E. coli* protein lipoyl ligase as reported earlier 26. Lipoylation of each E2‐derived domain was confirmed by FT‐MS.

#### Expression and purification of the E2o catalytic domain and its His375Ala and Asp374Ala variants

For expression of the CDo, DNA encoding residues 93–404 corresponding to the catalytic domain, subunit binding domain and linker connecting them in wild‐type E2o was synthesized and was cloned into pD451‐SR vector by DNA 2.0 Inc. (Menlo Park, CA, USA). DNA encoding CDo with His_6_ tag at the C‐terminal end was introduced into BL21 (DE3) cells (Novagen, San Diego, CA, USA), and cells were grown in LB medium supplemented with 35 μg·mL^−1^ of kanamycin at 37 °C overnight. 15 mL of the overnight culture was inoculated into 800 mL of the LB medium supplemented with kanamycin (35 μg·mL^−1^), and cells were grown to OD_600_ of 0.5–0.6 at 37 °C, and then, IPTG (0.5 mm) was added and the cells were grown for an additional 5 h at 37 °C. The cells were then washed with 50 mm KH_2_PO_4_ (pH 7.5) containing 0.15 m NaCl and were stored at −20 °C. The harvested cells were resuspended in 20 mm KH_2_PO_4_ (pH 7.5) containing 0.30 m NaCl, 1 mm benzamidine·HCl, 1 mm DTT, and two protease inhibitor cocktail tablets. Cells were treated with lysozyme (0.6 mg·mL^−1^) at 4 °C for 20 min and then with DNase I (NEB) and micrococcal nuclease (NEB) (1000 units of each) for an additional 20 min at 4 °C. Cells were disrupted by a sonic dismembrator with 10‐s pulse ‘on’ and 30‐s pulse ‘off’ for 5 min. The supernatant was centrifuged at 30 000 *g* for 20 min. The clarified lysate was loaded onto a 10 mL Ni‐Sepharose 6 Fast Flow column (GE Healthcare) pre‐equilibrated with 20 mm KH_2_PO_4_ (pH 7.5) containing 0.30 m NaCl, 1.0 mm benzamidine·HCl, and 10 mm imidazole. The column was first washed with 35 mm imidazole in the same buffer solution. The CDo was eluted with 150 mm imidazole. The fractions containing CDo were dialyzed against 2,000 mL of 20 mm KH_2_PO_4_, containing 0.45 m NaCl and 1.0 mm benzamidine**·**HCl for overnight at 4 °C. The CDo was concentrated by ultrafiltration with a 10‐kDa MW cutoff concentrating unit and was stored at −80 °C.

**Figure 2:**
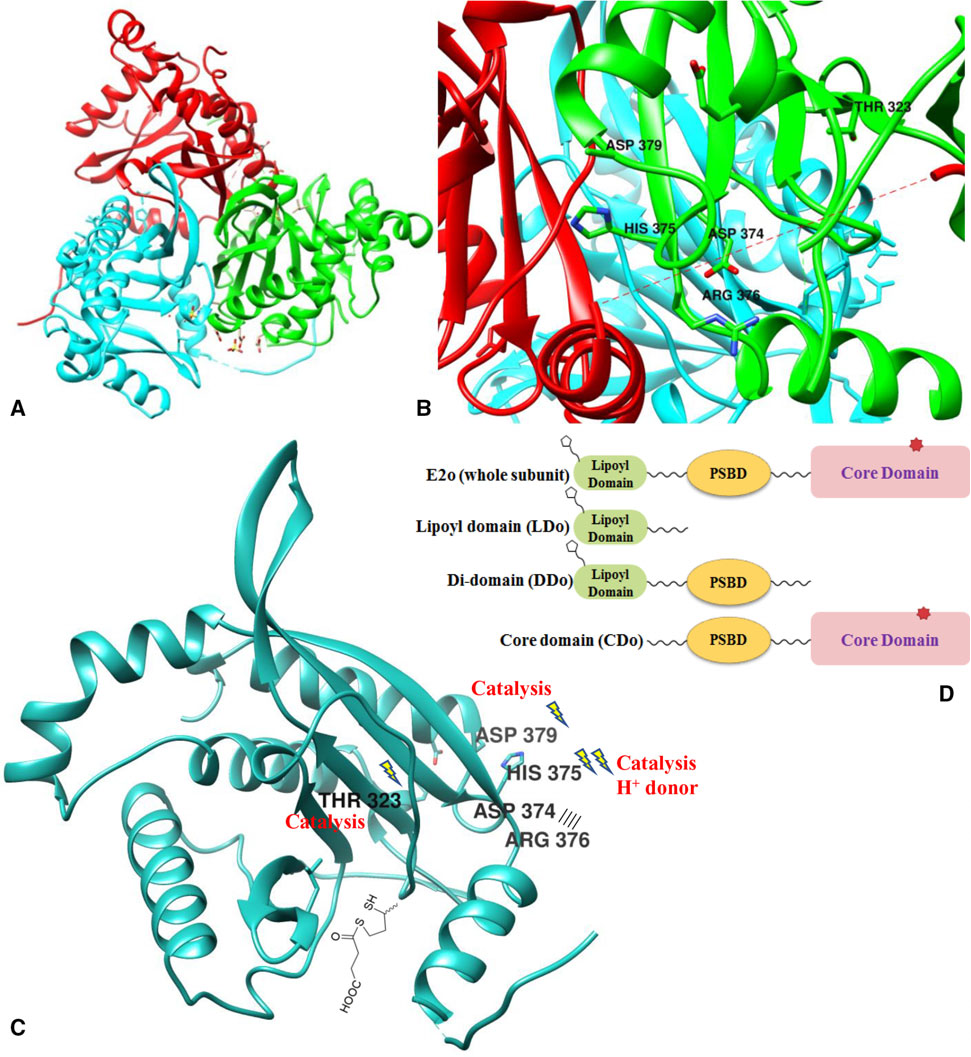
(A) Ribbon drawing showing the CDo trimer with each subunit in a different color. (B) E2o active site region at a dimeric subunit interface within a trimer showing positions of active site residues Thr323, Asp374, His375, Arg376, and Asp379 substituted in this study. Figures were prepared using the program UCSF Chimera 42 and the coordinates deposited in the PDB ID: http://www.rcsb.org/pdb/search/structidSearch.do?structureId=1c4t 7. (C) Ribbon diagram representing a single chain A of *t*E2o catalytic domain. (D) Diagrammatic representation of the E2o truncated proteins: A, unsubstituted E2o; B, lipoyl domain (E2o^1–105^); C, didomain (E2o^1–176^); D, core domain (E2o^93–404^).

For construction of Asp374Ala and His375Ala CDo variants, the pD451‐SR plasmid encoding CDo was used as a template, and the amplification primers such as 5′‐GCGCTGTC‐CTACGAT**GCG**CGTCTGATCGAT‐3′ (for His375Ala) and 5′‐CTGGCGCTGTCCTA‐C**G** **CG**CACCGTCTG ATC‐3′ (for Asp374Ala) and their complements were used for site‐directed mutagenesis (the mismatched bases are underlined, and mutated codons are shown in boldface type). The QuikChange Site‐Directed Mutagenesis Kit was used for single‐site substitution (Stratagene). The constructed domains are shown in Fig. 2D.

#### Expression and purification of E3

Expression and purification of E3 was carried out as reported earlier 27.

### Enzyme activity measurements

Overall OGDHc activity was measured in the reaction assay as reported earlier 23. For OGDHc assembly, E1o in 20 mm KH_2_PO_4_ (pH 7.0) containing 0.15 m NaCl was assembled with independently expressed E2o and E3 components with a mass ratio of E1o:E2o:E3 of 1 : 1 : 1 (μg : μg : μg) for 20 min at 25 °C. The reaction was initiated by OG (2 mm), or pyruvate (25 mm), or OV (45 mm), and CoA (0.13 mm).

In the OGDHc assay with CDo and LDo replacing E2o, the E1o (60 μg, 1.4 μm subunits) in 50 mm KH_2_PO_4_ (pH 7.0) was first preincubated with independently expressed CDo or CDo variants (60 μg, 4.9 μm subunits) and E3 (60 μg, 2.9 μm subunits) at a mass ratio of 1 : 1 : 1 (μg : μg : μg) at 25 °C. An aliquot with 5 μg of E1o (0.11 μm subunits) was then withdrawn and was mixed with varying concentrations of LDo in a cuvette containing all components needed for OGDHc assay. After 1 min of equilibration at 30 °C, the OGDHc reaction was initiated by the addition of OG (2 mm) and CoA (0.13 mm) as above.

### Site‐saturation mutagenesis on the His375 residue in the E2o core domain

The site‐saturation mutagenesis technique was used to create a library at the His375 position in the E2o core domain using a modified procedure for QuikChange Site‐Directed Mutagenesis Kit and using NNS primers 5′‐GGCGCTGTCCTACGAT**NNS**CGTCTGATCG**‐**3**′** (His375X) where N = A/T/G/C and S = G/C. The pCA24N‐E2o plasmid DNA was used as template. The library was created and transformed into *E. coli* BL21 (DE3)‐competent cells.

The library was plated onto an LB agar plate containing chloramphenicol antibiotic (50 μg·mL^−1^) to obtain single colonies. Approximately 270 single colonies were picked and inoculated into a 96‐well plate containing LB medium and chloramphenicol (50 μg·mL^−1^) to make master plates. The master plates were incubated at 37 °C at 250 rpm. Next, duplicate plates were constructed by inoculating (50× dilution) deep‐well plates that contained 1 mL LB media, chloramphenicol, D,L‐lipoic acid (0.3 mm), and IPTG (1 mm) for E2o. The deep‐well plates were incubated at 37 °C at 250 rpm. The cells were centrifuged at 1600 ***g**,* and the media were discarded. The cell pellets were stored −20 °C until use. The cell pellets were resuspended and lysed in a buffer containing KH_2_PO_4_ (20 mm, pH 7.5), NaCl (0.15 m), DTT (1 mm), protease inhibitor cocktail tablet, lysozyme (1 mg·mL^−1^), DNAse (0.1 mg·mL^−1^), and nuclease (1000U) and incubated for 1 h at 37 °C. The lysate was centrifuged twice at 1600 *g* for 30 min, and the clarified supernatant was used for the subsequent steps.

The E1o and E3 components were overexpressed separately each in 1L of culture medium following published protocols and lysed 27. The lysates of E1o, E2o, and E3 were reconstituted in a 96‐well plate (total volume 300 μL) in a buffer containing KH_2_PO_4_ (20 mm, pH 7.5), NaCl (0.15 m), MgCl_2_ (1 mm), and ThDP (0.1 mm) in a ratio of 1 : 2 : 1 as per OD_600 nm_/μL for 1 h and mixed well. The first column on the plate was the wild‐type OGDHc complex.

The overall activity was measured in a 200‐μL flat‐bottomed 96‐well plate containing 0.1 m Tris/HCl (pH 8.0), MgCl_2_ (1 mm), ThDP (0.1 mm), DTT (2.6 mm), NAD^+^(2.5 mm), CoA (0.13 mm), and OG (5–10 mm). NADH production was measured by the initial rate of reaction at 340 nm. The reaction was initiated using 60 μL of the assembled complex. The endpoint at 340 nm was measured with a plate reader (Spectramax^®^ M2 UV‐Vis) after 2, 5, 7, and 10 min.

Those E2 variants were chosen that had similar or higher activity than the wild‐type [(OD_340 final variant x_−OD_340__initial variant x_)/(OD_340__final variant_−OD_340__initial variant_)]. The candidates were rescreened, and the positive variants were sequenced.

The rescreening conditions were identical to the screening assay with a minor modification. First, identified clones were traced back to the master plate and restreaked on LB agar plates containing chloramphenicol (50 μg·mL^−1^). The first column was reserved for wild‐type, and two columns or 16 colonies from a single E2o‐variant were inoculated into a 96‐well plate containing LB medium and antibiotic. The false‐positives were discarded. The positive clones were sequenced, overexpressed, and purified following published procedures and as described above 24. Next, the purified E2o variants were assembled with purified E1o and E3 and the steady‐state kinetic parameters were determined.

### Fluorescence spectroscopy

To determine the dissociation constant of CoA and succinyl‐CoA with the E2o His375Trp variant, to the protein (0.24 mg·mL^−1^, concentration of active centers of 3.5 μm) in a mixture of 50 mm KH_2_PO_4_ and 50 mm Tris (pH 8.0), containing 0.1 m NH_4_Cl, 1 mm DTT, and glycerol (1% v/v), was added CoA (3–570 μm) or succinyl‐CoA (5–380 μm), and the fluorescence spectra were recorded after each addition at 25 °C using a Cary Eclipse spectrofluorimeter. The excitation wavelength was 295 nm, and the emission spectra were recorded in the range of 300–450 nm in 3‐mL quartz cuvettes. The *K*_d_ values for CoA were calculated using Eqn (1).

(1) $$\left(F_{o}-F_{i}\right)/F_{o}=\left(\Delta F_{\max}/F_{o}{\left[\text{CoA}\right]}^{n}\right)/\left(S_{0.5}^{n}+{\left[\text{CoA}\right]}^{n}\right)$$

where (*F*_o_ *−F*_i_)/*F*_o_ is the relative fluorescence quenching following the addition of CoA or suc‐CoA, *n* is the Hill coefficient, and for *n* = 1.0, the value of S_0.5_ is equal to *K*_d_.

### Reductive succinylation of the lipoyl domain by E1o and OG

For this experiment, the LDo (100 μm) was incubated with E1o (0.015 μm) in 35 mm (NH_4_)_2_CO_3_ (pH 7.5) containing 0.50 mm MgCl_2_ and 0.1 mm ThDP. The reaction was started by addition of 2 mm of OG. Aliquots of 10 μL were withdrawn at times of 5–600 s and were diluted into 1 mL of 50% methanol and 0.1% formic acid to stop the reaction, and then, the samples were analyzed by ESI FT‐MS. The fraction of succinylated LDo at different times was determined by taking a ratio of the relative intensity of the mass of the succinylated form to the total relative intensity (sum of unsuccinylated and succinylated LDo). The time dependence of this fraction was plotted, and the data were fitted to a single exponential (Eqn (2)) using Sigma Plot 10.0. The rate constant was calculated from the linear fit to the initial rate conditions.

(2) $$f=f_{o}+f_{1}\times \left(1-e^{-\mathrm{kt}}\right)$$

### Succinyltransferase reaction of the core domain and its His375Ala and Asp374Ala variants in the reverse direction

The following protocol outlines simultaneous detection of succinylated and unsuccinylated LDo forms when CDo was used as catalyst in the reverse reaction. The reaction mixture in 300 μL of the 35 mm NH_4_HCO_3_ (pH 7.5) contained the following: 40 μm LDo lipoylated *in vitro*; 0.10 mm TCEP to keep LDo in the reduced form; 0.15 mm succinyl‐CoA; and 0.05 μm CDo. The reaction was started by addition of CDo after 40 s of equilibration of the reaction assay and was conducted at 30 °C. Aliquots of 20 μL were withdrawn at times of 5–120 s and were diluted into 1 mL of 50% methanol and 0.1% formic acid to quench the reaction, and then, samples were analyzed by ESI FT‐MS. The fraction of succinylated LDo at different times was determined by taking a ratio of the relative intensity of the mass corresponding to the succinylated form to the total relative intensity (sum of intensities for the unsuccinylated and succinylated LDo). The time dependence of this fraction was plotted, and data were fitted to a single exponential (Eqn (1)) using Sigma Plot 10.0. The rate constant was calculated from the linear fit to the initial rate conditions.

## Results and Discussion

### Reductive succinylation of the lipoyl domain by E1o and OG is not the rate‐limiting step in the OGDHc reaction

Reductive succinylation of the lipoyl moiety covalently attached to the lipoyl domain on E2o is the final step involving ThDP‐bound covalent intermediates formed on the first E1o component of the OGDHc. It is a concomitant oxidation of the OG‐derived enamine and reductive acylation of E2o (Fig. 2, see Fig. 2D for C‐terminally truncated E2o proteins created by DNA manipulations and employed in these experiments). According to Fig. 1, the S8‐succinyldihydrolipoyl‐E2o is the product of the reductive succinylation of the lipoyl‐E2o by E1o and OG 28. Next, transfer of the succinyl group from the thiol ester of S8*‐*succinyldihydrolipoyl‐E2o to CoA takes place exclusively at the E2o core domain active centers (Fig. 2C), so there is no oxidation–reduction involved in succinyl transfer, rather it represents a conversion of one thiol ester to another.

**Figure 3:**
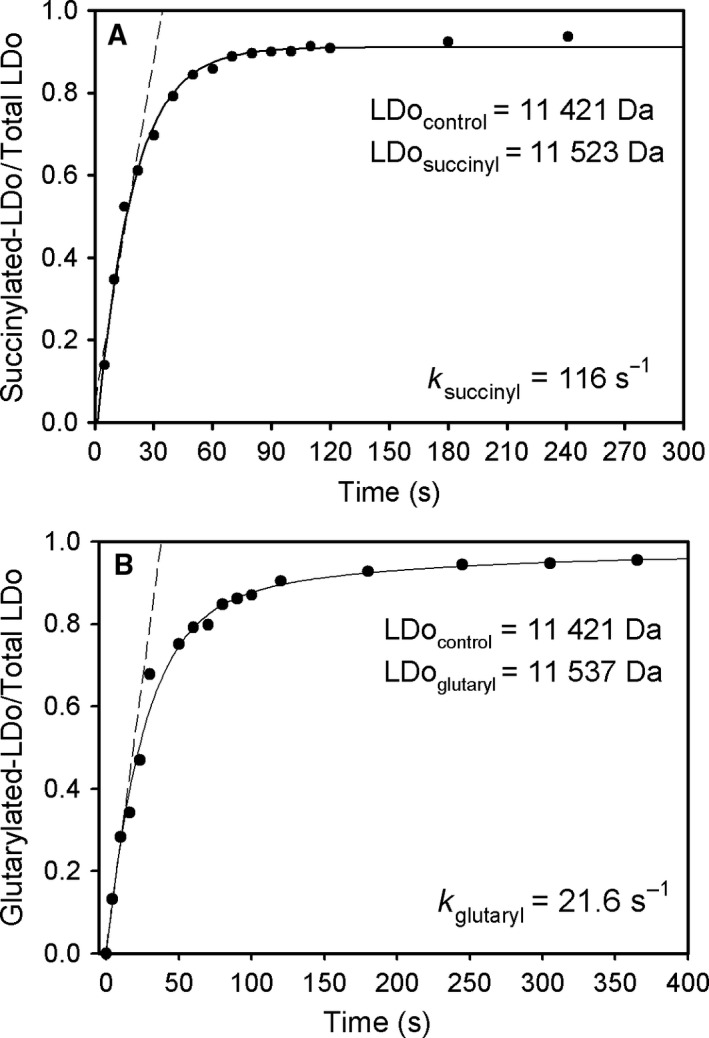
Time dependence of the reductive acylation of LDo by E1o and 2‐oxoglutarate or 2‐oxoadipate. (A) The reductive succinylation of the LDo by E1o and OG. LDo (100 μm), E1o (0.048 μm active centers), 0.1 mm ThDP, and 0.5 mm MgCl_2_ in 35 mm NH_4_ HCO_3_ (pH 7.5) was mixed with 2 mm OG. At time interval (5–240 s), aliquots of 10 μL were withdrawn and were quenched into 1 mL of 50% methanol and 0.1% formic acid. Samples were analyzed by FT‐MS. (B) The reductive glutarylation of the LDo by E1o and OA. The LDo (100 μm), E1o (0.075 μm active centers), 0.10 mm ThDP, and 0.5 mm MgCl_2_ in 35 mm NH_4_ HCO_3_ (pH 7.5) were mixed with 2.5 mm OA. Samples were analyzed by FT‐MS. The relative intensity of the acylated versus total LDo (sum of acylated and unacylated) was plotted versus time. The trace is a nonlinear regression fit to a single exponential rise to maximum (see Eqn (1)), and the dashed line represents a linear fit to initial rate conditions. The rate constants were calculated from the initial slope.

Earlier, we developed an appropriate model reaction 26 to study the rate of reductive acetyl transfer from *E. coli* pyruvate dehydrogenase (E1p) to the lipoyl domain of the dihydrolipoyl transacetylase (E2p), which maintains both the chemistry and the intercomponent communication due to its specific recognition of E1p 29, 30, 31. The method developed takes advantage of our ability to detect and quantify LD‐E2p and acetyl‐LD‐E2p simultaneously in the quenched reaction mixtures using Fourier transform mass spectrometry (FT‐MS). Analogously, the reductive succinylation of LDo by E1o and OG under steady‐state conditions here examined provides an appropriate model reaction to study the communication between the E1o and E2o components. The LDo employed in these studies was fully lipoylated *in vitro* by *E. coli* lipoyl protein ligase (see Materials and methods), and lipoylation was confirmed by FT‐MS 26. Two LDo forms were identified by FT‐MS: an LDo form with mass of 11421.2 Da that correlates well with the theoretical mass of LDo = 11420.4 Da, and an LDo form with mass of 11, 435.2 Da that differs from the previous form by 14 Da, possibly due to methylation of His_6_·tag as reported in the literature 32. Both LDo forms could be nearly completely reductively succinylated by E1o and OG (>90% succinylation) (Fig. 3A), leading to an LDo form with mass of 11523.2 Da (increase in mass by 102 Da on succinylation, the theoretical mass = 11522.4 Da), and LDo form with mass of 11537.2 Da. An average (based on three experiments) rate constant of 99 s^−1^ could be calculated from a steady‐state experiment for reductive succinylation of LDo (see Fig. 3A for a typical experiment) and was approximately twice as large as the *k*_cat_ of 48 s^−1^ determined from the overall OGDHc activity measurements in Table 2. These data indicate that with a sufficient amount of LDo in the reaction assay, the succinyl transfer from E1o to E2o is not a rate‐limiting step (48 s^−1^ is *k*_cat_ for overall activity vs. 99 s^−1^ for reductive succinylation). Importantly, the substrate analogues, 2‐oxoadipate (*k*_glutarylation_ = 22 s^−1^), 2‐oxovalerate (*k*_butyrylation_ **= 0.0084 s^−1^), and pyruvate (*k*_acetylation _= 0.048 s^−1^), could also be used by E1o as substrates for reductive acylation of LDo; however, their efficiency was lower than that with OG (see Fig. 3A for OG and 3B for OA). Finally, this model reaction is an important control to assure that the acyl group derived from 2‐oxoglutarate on E1o could be transferred to LDo before acyl‐CoA formation in the active centers of E2o and could lead to determination of the catalytic rate constants for acyl transfer.

**Table 2:** Kinetic parameters of the OGDHc assembled from E1o, E2o/indicated E2o variants, and E3 determined for NADH production in the overall and in E1o‐specific reactions

E2o Variant	OGDHc activity^a^ (μmol·min^−1^·mg^−1^)	*k*_cat_ (s^−1^)	*k*_cat_ */K*_m_ (m ^−1^·s^−1^)	E1o‐ activity^b^ (μmol·min^−1^·mg^−1^)	^*K*^_m,OG_^(m^ ^m^ ^)^	^*K*^_m,CoA_^(m^ ^m^ ^)^
E2o	13.7 ± 2.5 (100%)	48.0	1.07 × 10^6^	0.35 ± 0.01	0.05 ± 0.011	0.0204
Thr323Ala	6.2 ± 0.30 (45%)	21.7	0.48 × 10^6^	0.36 ± 0.01	0.095 ± 0.03	0.0233
Thr323Ser	5.9 ± 0.50 (43%)	20.7	0.46 × 10^6^	0.29 ± 0.03	ND	ND
Asp374Ala	0.28 ± 0.01 (2.1%)	0.98	0.02 × 10^6^	0.32 ± 0.02	0.077	ND
Asp374Asn	1.33 ± 0.03 (9.7%)	4.7	0.10 × 10^6^	0.40 ± 0.07		
His375Ala	0.29 ± 0.03 (2.1%)	1.0	0.02 × 10^6^	0.30 ± 0.01	0.082	ND
His375 Cys	0.13 ± 0.01 (1.0%)	0.46	0.01 × 10^6^	0.33 ± 0.04		
His375Asn	0.07 ± 0.01 (0.5%)	0.26	0.006 × 10^6^	0.44 ± 0.01		
His375Trp*	8.14 ± 0.4 (59.4%)	28.8	0.52 × 10^6^	0.33 ± 0.04	0.07 ± 0.02	0.0133
His375Gly*	1.76 ± 0.15 (12.6%)	5.76	0.18 × 10^6^	0.37 ± 0.08	0.04 ± 0.006	0.0206
Arg376Ala	7.7 ± 0.08 (56%)	27.0	0.60 × 10^6^	0.31 ± 0.04	0.14 ± 0.007	0.0198
Asp379Ala	1.3 ± 0.07 (9.5%)	4.6	0.10 × 10^6^	0.29 ± 0.01	ND	0.0211

^a^OGDHc was assembled from E1o, indicated E2o variants and E3 at a mass ratio of 1 : 1 : 1 (μg : μg : μg). The basis of using μg ratio instead of μm is to measure absolute amount of protein and be consistent as we use different constructs of the enzyme in the following experiments. Activity was calculated per mg of E1o. No OGDHc activity was detected for E1o or E3 in the absence of E2o.

^b^E1o‐specific activity of the assembled OGDH complexes was measured in a DCPIP assay and was calculated per mg of E1o 23. All assays were carried out in triplicates. The E2o* variants are the ones identified by site‐saturation mutagenesis.

### Residues Asp374 and His375 are the key catalytic residues in the active site of E2o

Next, the residue‐specific contribution to catalysis of succinyl‐CoA formation in the active center of the *E. coli* E2o was studied. Results of this study would be applicable to all E2o components due to high sequence identities reported for all E2o core/catalytic domains 13, 14, 15, 16, 17, 18, 19. Putative residues from the active center of the E2o involved in succinyl transfer and substrate binding were identified from the X‐ray structure of the truncated cubic core of the *E. coli* E2o, known as the E2o catalytic or core domain 13, 14 (Fig. 2A,B). The following E2o active center variants were created to determine their individual contributions to the catalytic efficiency of the OGDHc complexes assembled from E1o, E2o (or its active center variants), and E3: Thr323Ala, Thr323Ser, Asp374Ala, Asp374Asn, His375Ala, His375Cys, His375Asn, Arg376Ala, and Asp379Ala (Fig. 2B, Table 2). The activity of the assembled OGDHc variants was measured in the overall assay for NADH production and in an E1‐specific assay to study OG oxidation by OGDHc in the presence of the artificial electron acceptor 2,6‐dichlorophenolindophenol (DCPIP). The E1‐specific activity does not need the presence of the E2o and E3 components; however, substitutions in E2o could potentially affect assembly into OGDHc. As shown in Table 2, the Thr323 E2o substitutions to Ala or Ser do not significantly affect the NADH production as 45% (Thr323Ala E2o) and 43% (Thr323Ser E2o) of E2o activities were detected. From these results, it is unlikely that Thr323 in E2o is a catalytically important residue, contrary to the suggestion made based on the X‐ray studies 7. Similarly, the Arg376Ala E2o substitution led to 56% OGDHc activity remaining. In contrast, the Asp374Ala and Asp374Asn E2o substitutions revealed 2.1% and 9.7% of OGDHc activity remaining, respectively. Approximately, 9.5% of the OGDHc activity was detected for Asp379Ala‐substituted E2o (Table 2). The His375Ala, His375Cys, and His375Asn E2o substitutions at the highly conserved histidine 375 residue led to 2.1%, 1.0%, and 0.5% activity remaining, respectively, but did not abolish the OGDHc activity. These findings are in agreement with data reported from Rutgers earlier for substitutions at residue His339 in the E2p of the *E. coli* pyruvate dehydrogenase complex (PDHc), a position analogous to His375 in E2o 33. The activity of the *E. coli* PDHc was reduced to approximately 5.6% with the His399Ala E2p and 2.8% with the His399Cys E2p substitution, clearly indicating that cysteine substitution led to a lower activity of both E2os as compared with the Ala substitution 33. When OGDH complexes assembled with E2o and its variants were tested with pyruvate (25 mm) or OV (45 mm) as potential substrates for chemo‐enzymatic synthesis of acyl‐CoA analogues, no NADH production was detected, indicating that neither acetyl‐CoA nor butyryl‐CoA could be produced (data not presented). It is also noteworthy that neither the E1o‐specific activity of the reconstituted OGDH complexes, nor indeed the complex assembly itself, was affected by the indicated E2o substitutions (Table 2).

The following could be concluded: two residues, Asp374 and His375 from the E2o active center are catalytically most important, as substitution of either residue for Ala impaired E2o catalytic efficiency by 54‐fold, while a 107‐fold reduction was observed for the His375Cys E2o variant. Compared to the unsubstituted E2o, the His375Asn substitution lowered the catalytic efficiency by 178‐fold, while the Asp374Asn E2o lowered it by only 11‐fold. The rate retardation resulting from Ala substitution of all five active center residues here tested is ~125 000‐fold [this number represents the product of (*k*_cat_/*K*_m_)_wild‐type_/(*k*_cat_/*K*_m_)_variant_] for each Ala‐substituted variant. This number could be compared to the 30 000‐fold rate acceleration for acyl transfer between two aliphatic thiols reported by Hupe and Jencks in model systems 34.

### Functional importance of Asp374 and His375 in independently expressed E2o catalytic domain

**Figure 4:**
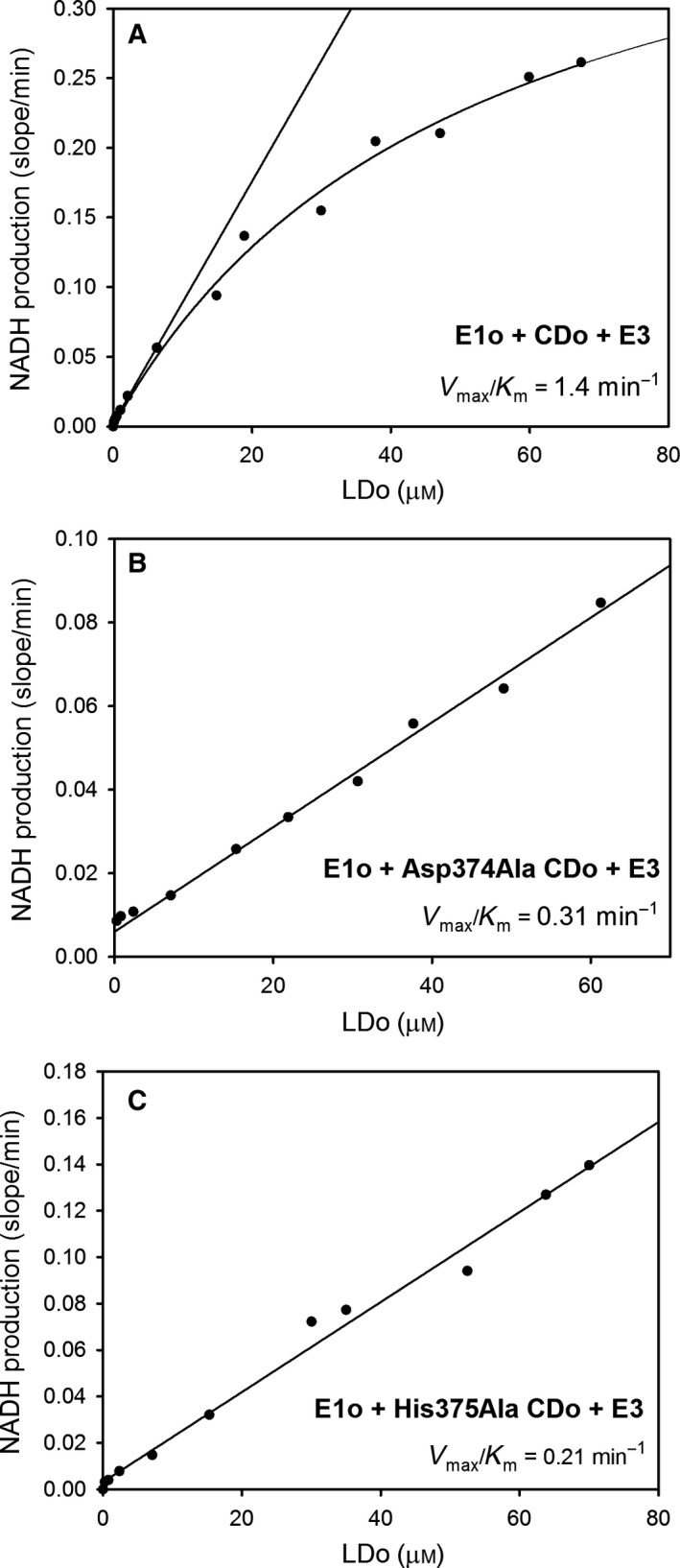
Dependence of the OGDHc activity on concentration of the lipoyl domain in the overall assay with CDo and LDo replacing E2o. (A) The E1o (3 μg, 0.028 μμ subunits), E3 (3 μg, 0.06 μμ subunits), and CDo (3 μg, 0.1 μμ subunits) were mixed at a mass ratio of 1 : 1 : 1 (μg : μg : μg) in 0.1 m Tris/HCl (pH 8.0) containing MgCl_2_ (1.0 mm), ThDP (0.2 mm), NAD ^+^ (2.5 mm), and different concentrations of the LDo (0–67.5 μμ) at 30 °C. After 5 min of equilibration, the reaction was initiated by OG (2 mm) and CoA (0.13 mm) and NADH production was recorded at 30 °C for 5 min. (B) The Asp374Ala CDo and LDo were used to replace E2o. (C) The His375Ala CDo and LDo were used to replace E2o. Condition of experiment for B and C was similar to that presented above for A.

**Figure 5:**
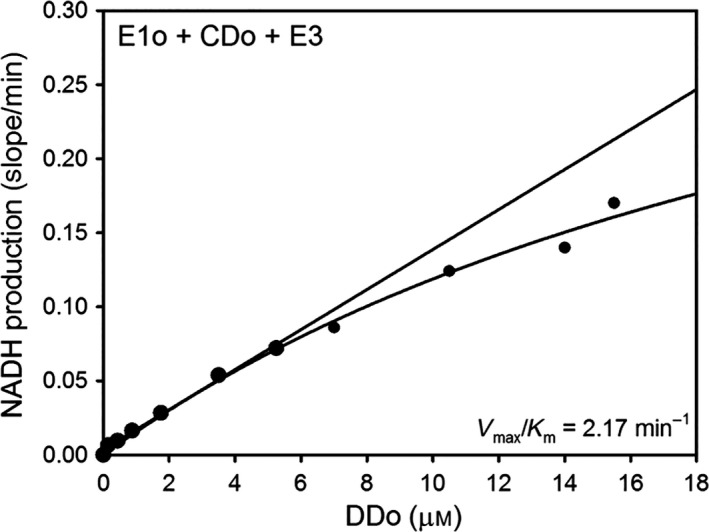
Dependence of the OGDHc activity on concentration of the E2o^1–176^ didomain in the overall assay with CDo and E2o^1–176^ didomain replacing E2o. For experimental conditions, see figure legend to Fig. 5.

An alternative approach to study coupling of the E2o active center with the peripheral E1o and E3 components was developed by us recently 15. It employs independently expressed E2o catalytic domain (CDo) in combination with a lipoyl domain source originated from E2o, such as lipoyl domain by itself (LDo) or C‐terminally truncated E2o proteins, such as E2o^1–176^ didomain, which consists of LDo, an E1o/E3 binding domain (peripheral subunit binding domain, PSBD) and flexible linkers connecting them (see Fig. 2D). For this approach, first, the functional competence of the independently expressed CDo in the overall OGDHc reaction needed to be proven. As shown in Figs 4 and 5, NADH production was detected on mixing of the CDo with varying concentrations of the LDo (0–70 μm) (Fig. 4A), or of E2o^1–176^ didomain (0 –16 μm) (Fig. 5), with E1o and E3 components added to complete the OGDH‐like complex. While the rate of NADH production was proportional to the concentration of the lipoyl domain source, no saturation could be reached with addition of either LDo or E2o^1–176^ didomain. Nor could saturation be reached with the Asp374Ala and His375Ala CDo variants (Fig. 4B,C). In the absence of saturation apparent in Figs 4 and 5, the values of the second‐order rate constants *k*_cat_ */K*_m_ could be calculated from the initial slope for NADH production: 0.124 × 10^6^ m ^−1^·s^−1^ (CDo + LDo domains), 0.192 × 10^6^ m ^−1^·s^−1^ (CDo + E2o^1–176^ didomain), 0.028 × 10^6^ m ^−1^·s^−1^ (CDo^Asp374Ala ^+ LDo), and 0.019 × 10^6^ m ^−1^·s^−1^ (CDo^His375Ala^ + LDo), in comparison with 1.07 × 10^6^ m ^−1^·s^−1^ for E2o by itself (Table 3). Again, the catalytic efficiency of the Asp374Ala and His375Ala CDo variants was 4.4‐ and 6.5‐fold lower than that for unsubstituted CDo. These results further affirm the contribution of Asp374 and His375 to E2o catalysis. Another important conclusion from this study is that the reaction intermediates could be transferred between the active centers of E1o, CDo, and E3 in the presence of lipoyl domain source in the reaction assay with no covalent bond linking CDo and lipoyl domain source. This is the second example from Rutgers, as similar findings were reported earlier for the *E. coli* E2p 15. In support of these striking conclusions, the following controls were performed: (a) No OGDHc activity was detected for LDo on its own in the absence of CDo; (b) negligible or no OGDHc activity could be detected for a mixture of E1o, E3, and CDo, but in the absence of LDo, thereby excluding any contamination from intrinsic wild‐type OGDHc. The results indicate that it will be sufficient to use independently expressed E2o domains for future active center engineering of E2o.

**Table 3:** The second‐order rate constants for NADH production in the overall assay where E2o was substituted by its indicated catalytic domain variants and lipoyl domain in comparison with E2o

E2o source	*k*_cat_ */K*_m_ (m ^−1^·s^−1^)^a^ forward direction	*k*_cat_ (s^−1^)^b^ reverse direction
E2o^a^	1.07 × 10^6^	n/a
CDo + LDo	0.124 × 10^6^	19.9
CDo^Asp374Ala^ + LDo	0.028 × 10^6^	1.86
CDo^His375Ala^ + LDo	0.019 × 10^6^	4.9

a Activity was measured in the NADH assay in the physiological direction.

b Activity was measured in the reverse direction. The concentrations of dihydro‐LDo were as follows: 35 μm for Asp374Ala CDo and 40 μm for both His375Ala CDo and for wild‐type CDo. For details on activity measurement see Materials and methods.

### Further evidence for the catalytic importance of Asp374 and His375 from the rate of succinyl transfer catalyzed by the E2o catalytic domain

To further substantiate the result of the His375Ala and Asp374Ala substitutions in the catalytic domain of E2o, an experiment was designed to study the succinyl transfer *per se* in the reverse reaction according to Eqn (3), thus enabling us to determine rate constants for the reaction taking place in the E2o active center exclusively. A mixture of dihydro‐LDo [LDo reduced by tris(2‐carboxyethyl)phosphine, TCEP] and CDo or its variants was reacted with succinyl‐CoA according to Eqn (3) where CDo or variants are the catalysts for the reaction.

(3) $$\begin{array}{cc} & \text{CDo}\text{succinyl}-\text{CoA}\\ & \;+\text{dihydro}-\text{LDo}\overset{\text{CDo}}{⇄}\text{succinyldihydro‐LDo}+\text{CoA} \end{array}$$

**Figure 6:**
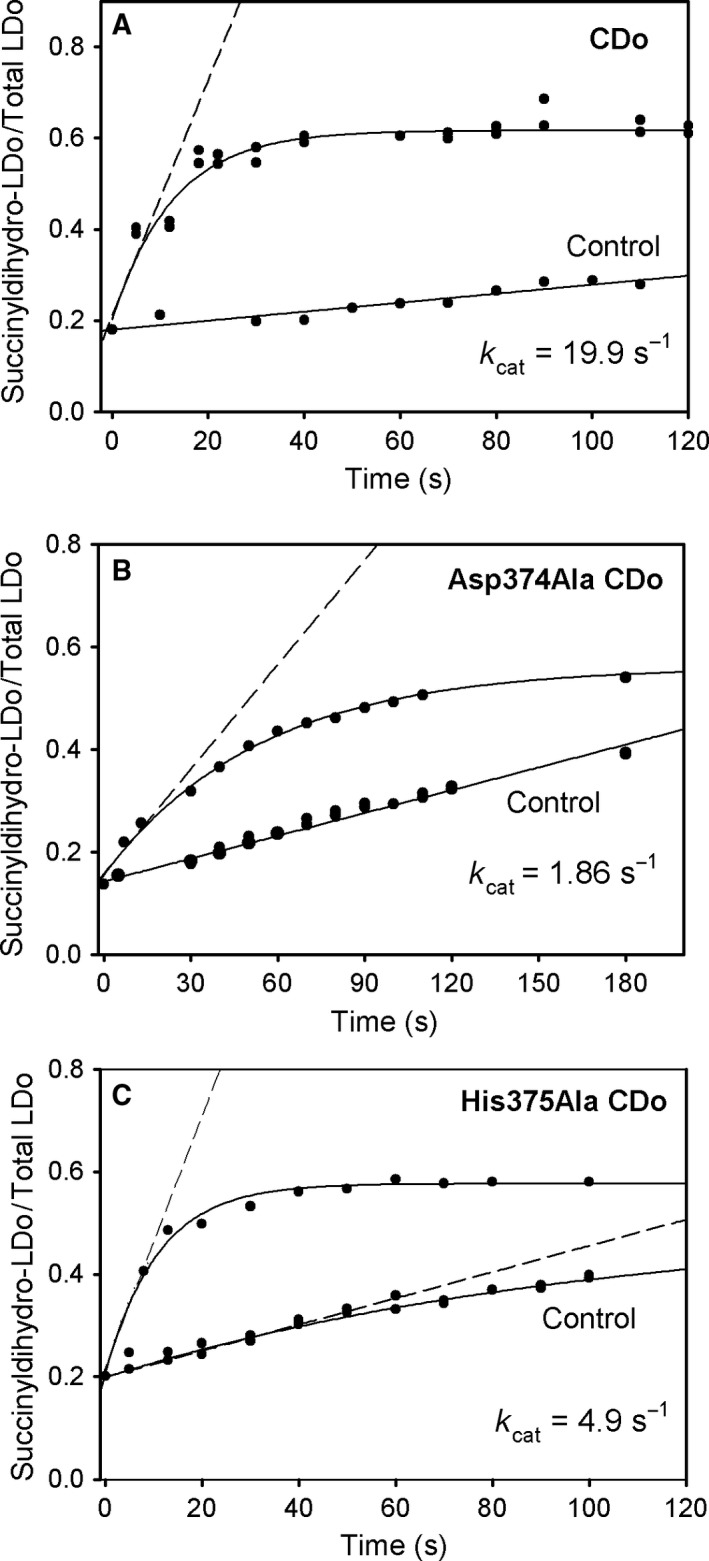
Kinetics of succinyldihydro‐LDo formation by CDo and variants in the reverse succinyltransferase reaction. (A) The dihydro‐LDo (40 μm), 0.10 mm TCEP, and 0.05 μm CDo in 35 mm NH_4_ HCO_3_ (pH 7.5) were mixed with 0.15 mm succinyl‐CoA. The reaction was stopped by addition of 50% methanol and 0.1% formic acid, and samples were analyzed for the presence of succinyldihydro‐LDo and dihydro‐LDo by FT‐MS. The relative intensity of succinyldihydro‐LDo versus total intensity (sum of succinyldihydro‐ and dihydro‐LDo) was plotted versus time. A control experiment was performed in the absence of CDo. (B) Progress curves of succinyldihydro‐LDo formation by Asp374Ala CDo. The reaction assay in 0.30 mL of 35 mm NH_4_ HCO_3_ (pH 7.5) contained the following: 35 μm dihydrolipoyl‐LDo, 0.35 mm TCEP, and 0.15 mm succinyl‐CoA. After 40 s of pre‐incubation, the reaction was started by addition of 0.10 μm Asp374Ala CDo. (C) Progress curves of succinyldihydro‐LDo formation by His375Ala CDo. The reaction assay was similar to that in B except of 40 μm dihydrolipoyl‐LDo was used. The traces are the nonlinear regression fit to a single exponential rise to maximum, and the dashed line represents a linear fit to initial rate conditions. For rate constants calculation in B and C, the slope in the control experiment was subtracted from that in the experiment.

Direct measurement of the masses of dihydro‐LDo and succinyldihydro‐LDo by FT‐MS was used to quantify the rate of succinyl transfer from succinyl‐CoA to the dihydro‐LDo. At concentrations of dihydro‐LDo (40 μm), succinyl‐CoA (0.15 mm), and CDo (0.05 μm), the dihydro‐LDo is a substrate, CDo is a catalyst, and succinyldihydro‐LDo is a product in Eqn (3). The reaction was stopped at different times (5–120 s) by diluting into 50% methanol and 0.1% formic acid, and the samples were analyzed by FT‐MS. The ratio of succinyldihydro‐LDo / total LDo (the sum of succinyldihydro‐LDo and dihydro‐LDo) was plotted versus time (Fig. 6A). As seen from Fig. 6A, approximately 60% of dihydro‐LDo is converted to succinyldihydro‐LDo with a rate constant of 19.9 s^−1^ as calculated from a linear fit to the initial rate of succinyldihydro‐LDo formation (Table 3). It should be noted that some time‐dependent formation of succinyldihydro‐LDo was detected by FT‐MS even in the absence of CDo in the reaction assay. While this nonenzymatic reaction was insignificant with unsubstituted CDo, the slope of this nonenzymatic reaction was taken into account when rate constants were calculated for the His375Ala and Asp374Ala CDo variants (Fig. 6B,C). As seen in Fig. 6 and Table 3, the rate constant of 1.86 s^−1^ (Asp374Ala) and of 4.9 s^−1^ (His375Ala) was 11 times and 4 times smaller, respectively, compared to the rate constant of 19.9 s^−1^ for unsubstituted CDo. A ratio of succinyldihydro‐LDo/total LDo of ~ 0.55–0.60 (reproducible with wild‐type and variant CDos in Fig. 6) was observed. The data above, and presented in Table 3, support the conclusion that Asp374 and His375 are important residues for succinyl transfer in both directions, as they need to be, while also suggesting an equilibrium constant near unity for the reaction in Eqn (3).

### Site‐saturation mutagenesis on the E2o active center His375

To gain further insight into the possible role of His375, we carried out site‐saturation mutagenesis on this residue and screened for possible variants that display OGDHc activity.

**Figure 7:**
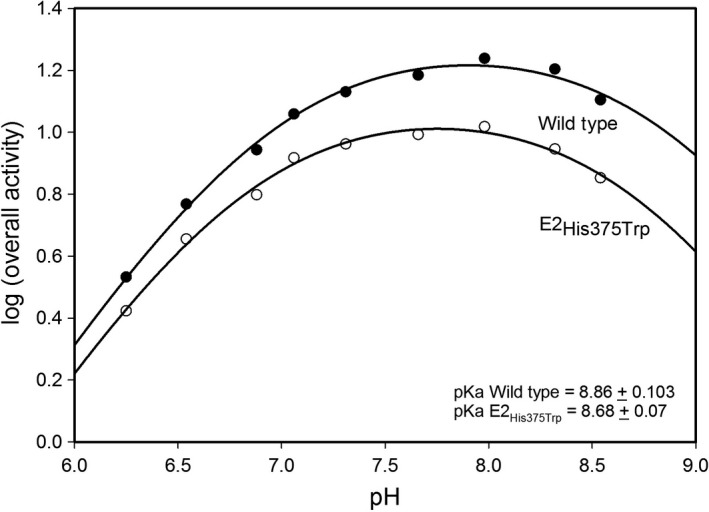
pH dependence of the OGDHc activity for wild‐type E2o and His375Trp E2o variant. The E1o (3 μg, 0.028 μμ subunits), E3 (3 μg, 0.06 μμ subunits), and wild‐type E2o (3 μg, 0.1 μμ subunits) or His375Trp E2o were mixed at a mass ratio of 1 : 1 : 1 (μg : μg : μg) in the reaction assay containing 0.05 m Tris/HCl, 0.05 m KH_2_ PO_4,_ MgCl_2_ (1.0 mm), ThDP (0.2 mm), and NAD ^+^ (2.5 mm) with pH of the reaction assay varied from 6.28 to 8.5. The reaction was initiated by CoA (0.13 mm) and OG (2 mm) and NADH production was recorded at 30 °C for 1 min. The values of activity were plotted to a curve defined by one ionizing group according to Eqn (4). (4)$\log\left(\text{activity}\right)=\log\left({\text{activity}}_{\max}\right)-\log\left(1+10^{\mathrm{pK}1-x}\right)$ where *x* is the value of pH.

The following His375 variants were identified: His375Trp E2o with a significant retention of the overall NADH activity (60%); and His375Gly E2o with significantly reduced NADH activity (13%) compared to unsubstituted E2o (Table 2). Steady‐state kinetic experiments revealed an approximately twofold reduction in *k*_cat_ for the His375Trp E2o (29 s^−1^) and about eightfold reduction for the His375Gly E2o (6 s^−1^) compared to E2o (48 s^−1^) (Table 2). No significant changes in values of *K*_m_ with respect to OG or CoA were detected for the variants in comparison to the unsubstituted E2o (Table 2). To further test whether His375 participated as a general acid–base catalyst, the pH dependence of the overall NADH activity of the His375Trp E2o and of the unsubstituted E2o, both assembled with the E1o and E3 components, was compared and revealed no difference in their shapes. The p*K*_app_ for the alkaline limb of the curve was 8.8 for E2o and 8.7 for the His375Trp E2o variant (Fig. 7). This parallel behavior of the pH dependence with His or Trp effectively rules out an acid–base role for His375.

**Figure 8:**
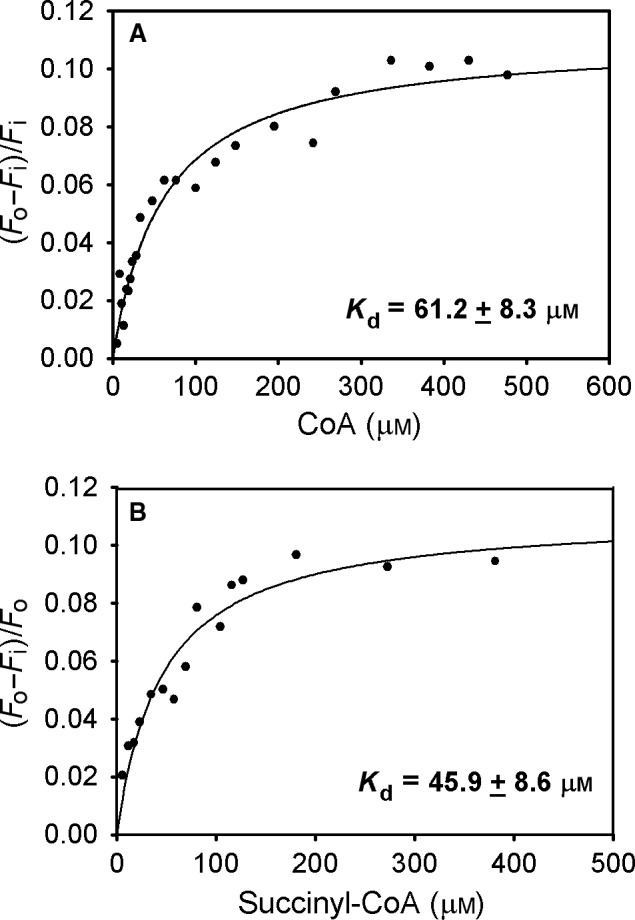
Quenching of the intrinsic fluorescence of the His375Trp E2o by CoA and succinyl‐CoA. (A) The top panel shows the dependence of the relative fluorescence quenching on the concentration of CoA. His375Trp E2o (0.24 mg mL^−1^, concentration of active centers of 3.5 μm) in a mixture of 50 mm KH_2_ PO_4_ and 50 mm Tris (pH 8.0) containing 0.15 m NH_4_Cl, 1 mm DTT, and 1% glycerol was titrated with CoA (3–570 μm). (B) The bottom panel shows the dependence of the relative fluorescence quenching on the concentration of succinyl‐CoA (5–380 μm). Data points in A and B were fit to Hill Eqn (1); the lines are the regression fit trace.

As there is no tryptophan residue in the E2o active center, we could also utilize the intrinsic fluorescence of the newly installed Trp in position 375 of the His375Trp E2o variant to determine the nearly equal *K*_d_ of CoA (61.2 μm) and succinyl‐CoA (47 μm) from quenching of fluorescence on addition of either compound (Fig. 8). As control, the E2o experienced no fluorescence quenching, thus also confirming substitution at the active center. In view of the modest decrease in *k*_cat_ */K*_m_ with the Trp substitution at position 375, these *K*_d_ values are appropriate for the unsubstituted E2o, and indicate similar binding affinity of E2o for CoA and succinyl‐CoA, where both succinyl and CoA moieties appear to contribute to binding.

## Conclusion

This paper is directed to an elucidation of the fundamental mechanism of the transthioesterification reaction carried out by the E2o of the *E. coli* OGDHc that would be applicable to all E2o components due to high sequence identities reported for the E2 catalytic domains 13, 14, 15, 16, 17, 18, 19. Important classes of enzymes carrying out similar reactions include inteins in expressed protein ligation 35 and many reactions on the polyketide pathways 36. Based on our studies, the following important results emerged.

**Figure 9:**
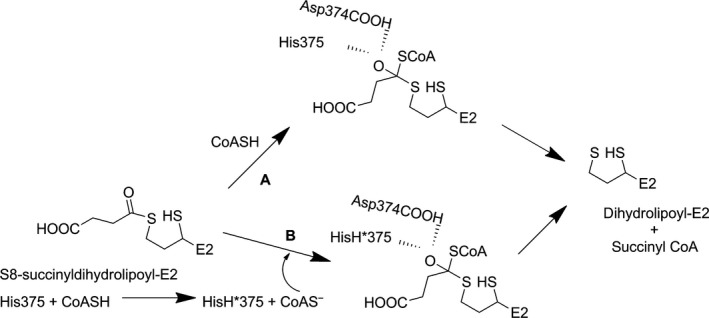
Likely mechanisms of succinyl transfer from dihydrolipoamide‐E2o to CoASH. Pathway A: direct attack by the conjugate base thiolate anion of CoASH assuming a low p*K*_a_ for the CoASH, or by the thiol form itself. Pathway B: initial proton transfer from CoASH to His375 forming the conjugate base CoAS ^−^, which is the attacking agent. Both pathways then proceed by an oxyanionic tetrahedral intermediate consistent with model studies 34.

- Identification of catalytically important Asp374 and His375 residues of the E2o catalytic domain and the rate acceleration provided by these residues. The other residues tested displayed much smaller contribution to catalysis. Using the E2o variants substituted by alanine at the putative catalytic center for transthioesterification, NADH production as a measure of the complex activity indicated that the five residues tested accounted for 2.23 (Thr323Ala), 54 (Asp374Ala and His375Ala each), 1.78 (Arg376Ala), and 11 (Asp379Ala)‐fold rate accelerations for a total of ˜125 000‐fold. This is the first identification of partial catalytic rate constants for this important reaction and raises the question: Which likely mechanism is consistent with the results?
- A closer look at the transthioesterification mechanism was provided by the use of independently expressed E2o domains (LDo, E2o^1–176^, and CDo) that allowed us to conclude that both Asp374 and His375 are important residues for succinyl transfer in both the physiological and in the reverse direction.
- The rate of reductive acylation of LDo by a number of substrate analogues (2‐oxoadipate, 2‐oxovalerate and pyruvate) signals that communication between E1o and E2o is not fatally compromised by the alternative substrates. The rate constant of 99 s^−1^ for reductive succinylation of the LDo by E1o and OG (the rate constant for the reaction starting with free enzyme and culminating in reductive succinylation of E2o, resulting in formation of S8‐succinyldihydrolipoyl‐LDo) compared to the *k*_cat_ of 48 s^−1^ (NADH formation by OGDHc).
- These results raise the question: Which likely mechanism of transthiolacylation is consistent with the experimental findings? Based on numerous precedents, there are two prominent likely mechanisms to account for the transfer of acyl group between two thiols depicted by the step characterized by the rate constants *k*_*6*_ and *k*_*‐6*_ in Fig. 1. A general acid–base mechanism would suggest that the His375 converts the thiol of the attacking nucleophile (CoASH) to a thiolate anion (CoAS^‐^) as depicted on pathway B in Fig. 9. The activated thiolate then attacks the carbonyl atom of the succinyldihydrolipoyl‐E2 to form a tetrahedral intermediate, which is stabilized by the hydroxyl side chain of Thr323 14. This notion was long accepted based on analogy with a mechanism developed for chloramphenicol acetyltransferase (CAT) 20, 21, 37. When the analogous His195 in chloramphenicol acetyltransferase was replaced by Ala, a 9 × 10^5^‐fold decrease in *k*_cat_ and virtually no change in *K*_m_ was observed, that is, a (*k*_cat_/*K*_m_)_wild‐type CAT_/(*k*_cat_/*K*_m_)_His195Ala CAT_ = 500 000 15. In contrast, the (*k*_cat_/*K*_m_) _unsubstituted E2o_/(*k*_cat_/*K*_m_) _His375Ala _= 54. This difference strongly suggests different functions for the highly conserved active center histidine on the two enzymes.

As a ‘gold standard’ for acid–base catalysis by a histidine side chain, we also present comparison with the much studied His64 at the catalytic triad of the subtilisin family of serine proteases, where the His64Ala substitution led to a reduction of 10^6^‐fold in *k*_cat_/*K*_m_, a number similar to that observed for CAT 38.

As an alternative to acid–base catalysis, for a protein with a p*K*_a_ for the cysteine thiol group near pH 7.0, one could envision direct attack by a thiolate anion on the thiol ester carbon, forming an essentially symmetrical tetrahedral oxyanionic intermediate, in which the central carbon atom is flanked by two C–S bonds, where either C–S bond has nearly equal probability for cleavage (Fig. 1, lower left and Fig. 9 pathway A) 34. We suggest that the nearly equal magnitude of the rate acceleration provided by the His375 and the Asp374 residues, more likely, reflects their roles in stabilizing the oxyanion by two hydrogen bonds (Fig. 9, pathway A), in other words creating a so‐called ‘oxyanion hole’. It had been demonstrated some years ago that in subtilisin, where a putative transition state stabilizing oxyanion hole is created by one main chain hydrogen bond donor and one side chain hydrogen bond donor (Asn155), substitution of Asn155 to Leu had small effect on the *K*_m_ but reduced the *k*_cat_ by a factor quoted as 200‐ to 300‐fold 39. This is very similar in magnitude to the *k*_cat_/*K*_m_ reductions observed (54 for each) with the Asp374Ala and His375Ala substitutions reported in this study (again no change in *K*_m_ was observed). Our results also concur with homology modeling studies between the enzymes chloramphenicol acetyl transferase and dihydrolipoamide acetyl transferase about existence of a His‐Asp‐Gly consensus in the catalytic core which is likely to play an important role 21.

While well accepted, it has been difficult to obtain experimental proof for the existence of an oxyanion in solution; as an example, proton inventory solvent kinetic isotope effect studies, a very subtle method, failed to do so 40.

The proposed hydrogen bond donation by Asp374 and His375 was tested by studying the alternative Asp374Asn substitution in E2o that allows hydrogen bond donation similar to His375 (Table 2). The His375Asn substitution led to 4.0‐fold further reduction in the E2o catalytic efficiency (to 0.5% of the remaining activity) compared to the His375Ala substitution (to 2.1%). In contrast, the Asp374Asn substitution partially rescued the E2o catalytic efficiency (9.7%) compared to Asp374Ala E2o (2.1% activity) and suggested that Asp374 is more likely to be involved in hydrogen bond formation. The result also raises an important implication that the carboxyl group of Asp374 has an unusually elevated p*K*_a_ as only in the conjugate acid form could it serve as a hydrogen bond donor. As this step appears to be part of rate limitation, we further speculate that the alkaline limb of the two activity–pH profiles (parallel for His375 or Trp375), and the deduced p*K*_a_ of 8.7–8.8 pertains to a highly perturbed Asp374 environment.

The His375 residue appears to be more sensitive to substitutions, suggesting a more complicated scenario. We therefore carried out site‐saturation mutagenesis on this residue and screened for substitution‐tolerant active variants. Informatively, the His375Trp and His375Gly variants were found to have intermediate activity (60% and 13%, respectively). Steady‐state kinetic analysis of the enzymes harboring these substitutions revealed only modest changes in *K*_m_. But, the twofold decrease in the *k*_cat_ for His375Trp E2o compared to the wild‐type E2o is inconsistent with the earlier suggestion that His375 acts as a proton donor for protonation of the leaving group. Our results are consistent with studies by McLeish's laboratory on the ThDP‐dependent enzyme benzoylformate decarboxylase, where similar changes were observed on site‐saturation mutagenesis studies based on the active site His281, by finding that the His281Phe and His281Trp variants displayed significant activity 41, and also arguing strongly against acid–base catalytic activity. Additional argument against an acid–base role of His375 is provided by the similar activity–pH profiles of unsubstituted E2o and its His375Trp variant. Given the results with the His375Trp E2o variant, we need to be cautious about assigning a role to the His375 side chain: rather than a direct participation in a hydrogen bond as suggested in Fig. 9, perhaps the imidazole ring provides a physical barrier protecting the hydrogen bonding unit, a role that could also be played by the indole side chain of Trp. Of course, there is also the possibility that the indole NH of Trp375 is the hydrogen bond donor for oxyanion stabilization.

Using a variety of experiments, the accumulated evidence allowed us to conclude that the rate‐limiting step on OGDHc is succinyl transfer to CoA in the E2o active center. In contrast, on the *E. coli* PDHc, the rate‐limiting step is the initial addition of substrate to the E1p component forming the first covalent predecarboxylation intermediate 26. The results also provide crucial information for further engineering of the E2o component for producing a variety of acyl‐CoA analogues.

## Author contributions

JC performed the biochemical and molecular biology experiments. NSN performed and analyzed the mass spectrometry experiments. JC, NSN, EF, and FJ wrote the article. FJ and EF directed the study.
